# Improving the efficacy of therapeutic angiogenesis by UTMD-mediated Ang-1 gene delivery to the infarcted myocardium

**DOI:** 10.3892/ijmm.2015.2226

**Published:** 2015-05-28

**Authors:** QING DENG, BO HU, SHENG CAO, HONG-NING SONG, JIN-LING CHEN, QING ZHOU

**Affiliations:** Department of Ultrasound Imaging, Renmin Hospital of Wuhan University, Wuhan, Hubei 430060, P.R. China

**Keywords:** targeted microbubble, myocardial infarction, angiogenesis, intercellular adhesion molecule-1, ultrasound irradiation

## Abstract

This study aimed to verify the feasibility and efficacy of ultrasound-targeted microbubble destruction (UTMD)-mediated angiopoietin-1 (Ang-1) gene delivery into the infarcted myocardium. Microbubbles carrying anti-intercellular adhesion molecule-1 (ICAM-1) antibody were prepared and identified. The microbubbles carrying anti-ICAM-1 antibody selectively adhered to the interleukin (IL)-1β-stimulated ECV304 cells and to the ischemic vascular endothelium, and the infarct area was examined to evaluate the targeting ability of ICAM-1 microbubbles *in vitro* and *in vivo*. The intravenous administration of the Ang-1 gene was carried out by UTMD in rabbits with acute myocardial infarction (AMI). The rabbits were divided into the control (no treatment), non-targeted microbubble destruction (non-TMB) and the ICAM-1 TMB (TMB) group. Gene delivery by direct intramyocardial injection (IMI) served as a reference. Two weeks later, regional myocardial perfusion and cardiac function were evaluated by echocardiography, and Ang-1 gene-mediated angiogenesis was assessed histologically and biochemically. The results revealed that the ICAM-1-targeted microbubbles selectively adhered to the IL-1β-stimulated ECV304 cells *in vitro* and to the ischemic vascular endothelium in the infarct area of the rabbits with AMI. Two weeks after the delivery of the Ang-1 gene, compared with the non-TMB group, left ventricular function and myocardial perfusion at the infarct area had improved in the TMB and IMI group (p<0.01). Ang-1 gene expression was detectable in the non-TMB, TMB and IMI group, while its expression was higher in the latter 2 groups (all p<0.01). The microvascular density (MVD) of the infarct area in the non-TMB, TMB and IMI group was 65.6±4.4, 96.7±2.1 and 100.7±3.6, respectively (p<0.01). The findings of our study indicate that UTMD-mediated gene delivery may be used to successfully deliver the Ang-1 gene to the infarcted myocardium, thus improving the efficacy of therapeutic angiogenesis. This may provide a novel strategy for future gene therapy.

## Introduction

Ultrasound-mediated microbubble fragmentation technology, which uses high-intensity ultrasound exposure to cause ‘sonoporation’ in cells and results in the uptake of genes, has become a relatively safe and promising method of gene delivery in recent years. Studies on ultrasound-targeted microbubble destruction (UTMD)-mediated angiogenic gene delivery for the treatment of ischemic heart disease have attracted much attention (1,2). Fujii *et al* (3) demonstrated that repeated exposure to UTMD promoted angiogenesis in the infarcted rat heart without causing cardiac injury. Yuan *et al* (4) found that the direct intramyocardial injection (IMI) of the hepatocyte growth factor (HGF) gene in conjunction with microbubbles enhanced angiogenesis by approximately 10.7-fold in dogs with myocardial infarction. However, at present, this gene transfection technique has failed to obtain satisfactory results in pre-clinical or clinical studies when the gene was administrated intravenously, but not by direct IMI (5–7). This low efficacy may be caused by limitations of the technique or the wide distribution of lipid-shelled microbubbles in the body (8,9). As a result, the concentration and population of microbubbles in the area of interest is not high enough to achieve biological effects. Therefore, the enhancement of the microbubble population or the density at the target site is essential in order to improve the efficacy of UTMD via intravenous administration. In a previous study, Browning *et al* (9) found that the efficacy of ultrasound-mediated gene transfection and the contrast agent, SonoVue, improved 3-fold by using larger gauge needles to infuse more bubbles in rats, which indicated that along with the increase in the number of microbubbles, the biological effects increased as the bioeffects of cavitation were thought to be the main mechanism of transfection (9). Their study focused on the total number of microbubbles infused *in vivo* into the circulation in animals. However, the suitable needle size may vary from large to small animals, and may not vary that greatly between humans. Thus, we hypothesized that the enhancement of the local microbubble population at the site of interest rather than the greater number of total microbubbles infused into the circulation would also improve the efficacy of ultrasound-mediated gene transfection.

Currently, the targeted delivery technique, which may enhance the microbubble population and density in the target organ mainly involves 3 aspects: i) ultrasound-exposure mediated microbubble destruction; ii) microbubbles loaded with a tissue-specific ligand for the area of interest; iii) the encapsulation of a gene or drug into the microbubbles and releasing them by ultrasound triggering into the target tissue (10). In this study, we combined a tissue-specific ligand with microbubbles in an aim to enhance the local microbubble population in the infarcted myocardium, and applied ultrasound irradiation for controlled gene release with high efficacy.

It has been demonstrated that impaired endothelial cells in the ischemic region overexpress intercellular adhesion molecules (ICAMs), mainly ICAM-1 (11). Therefore, in this study, ICAM-1 was selected as a ligand to strengthen the targeting ability of microbubbles in the infarcted myocardium. The therapeutic gene introduced was angiopoietin-1 (Ang-1) gene, as its expression product is a protein molecule which plays an important role in the process of angiogenesis, and its effects are more long-term than those of vascular endothelial growth factor (VEGF) (12). The Ang-1 gene also inhibits endothelial cell apoptosis, promotes vessel matuarion, maintains the stability of blood vessels and antagonizes the vascular permeability caused by endothelial growth factors, ultimately attenuating ventricular remodeling and cardiac dysfunction due to the lack of myocardial cells (13,14). Based on these data, in this study, we aimed to construct a microbubble loaded with anti-ICAM-1 monoclonal antibody as a Ang-1 gene carrier, and to determine the ability of the targeted microbubbles to improve the efficacy of therapeutic angiogenesis via ultrasound exposure in the infarcted myocardium in comparison to common non-targeted microbubbles.

## Materials and methods

### Preparation and appraisal of ICAM-1-targeted microbubbles

Our study was carried out at the Medicine and Virology Laboratory of the College of Life Science in Wuhan University (Wuhan, China). SonoVue microbubbles were diluted with normal saline as per the manufacturer’s instructions (Bracco, Milan, Italy). The mouse anti-human ICAM-1 antibody (jm-6140R; Jingmei Biology Co., Shenzhen, China) marked by fluorescein isothiocyanate (FITC; Sigma, St. Louis, MO, USA) was mixed with phosphate-buffered saline (PBS) at a 1:50 volume ratio. Microbubble suspension solution was mixed with the antibody at a 2:1 volume ratio and incubated for 2 h at 4°C. Afterwards, the unbounded free antibody was rinsed off with PBS solution and the upper foam was re-suspended.

It was considered that the FITC-labeled ICAM-1 antibody had successfully incorporated into the SonoVue microbubbles, as indicated by bright green fluorescence at the fringe of the microbubble surface under a fluorescence microscope (Olympus, Tokyo, Japan) during the experiment. The efficacy of the microbubble and antibody combination was determined using a flow cytometer (Beckman Coulter, Brea, CA, USA).

### Determination of the targeting ability of ICAM-1-targeted microbubbles for inflammatory endothelial cells

Human vascular endothelial ECV304 cells (China Center for Type Culture Collection, Wuhan, China) with or without human interleukin-1β (IL-1β) stimulation were divided into 5 groups as follows:

### Group 1: IL-1β stimulation + ICAM-1-targeted microbubbles (n=5)

The ECV304 cells were labeled with 3,3′-dioctadecyloxacarbocyanine perchlorate (DiO; Molecular Probes, Sigma) and added to recombinant IL-1β solution (PeproTech, Rocky Hill, NJ, USA). The final concentration of the IL-1β solution was 100 U/ml, as previously described (15). The ECV304 cells were stimulated for 5 h in order to highly express ICAM-1. The ICAM-1-targeted microbubbles were added to the IL-1β stimulated ECV304 cells (200 *µ*l/well) and placed in 5% CO_2_ at 37°C for 30 min of incubation.

### Group 2: No stimulation + ICAM-1-targeted microbubbles (n=5)

Normal ECV304 cells (not stimulated with IL-1β) were added to 200 *µ*l of the ICAM-1-targeted microbubbles and incubated in 5% CO_2_ at 37°C for 30 min.

### Group 3: IL-1β stimulation + common microbubbles (n=5; negative control)

The IL-1β-stimulated ECV304 cells were added to 200 *µ*l FITC-labeled microbubbles (without anti-ICAM-1 antibody) and incubated in 5% CO_2_ at 37°C for 30 min.

### Group 4: No stimulation + common microbubbles (n=5)

Normal ECV304 cells were added to 200 *µ*l FITC-labeled microbubbles (without anti-ICAM-1 antibody) and incubated in 5% CO_2_ at 37°C for 30 min.

### Group 5: Blank control (n=5)

IL-1β-stimulated ECV304 cells without any reagent and incubated in 5% CO_2_ at 37°C for 30 min.

The cells of each group were exposed to ultrasound following incubation. The probe was placed under the 6-well plate at a distance of 3–5 mm, the irradiation frequency ranged was 0.5–2 MHz with a continuous wave (UGT2007 ultrasound irradiation machine; Ultrasonic Research Institute, Chongqing Medical School, Chongqing, China). The intensity of ultrasound exposure was set at 1.5 W/cm^2^ and the time was 30 sec, as previously described (16,17).

The determination of the adhesion of the ICAM-1-targeted microbubbles to the ECV304 cells carried out by measuring the fluorescence intensity under a fluorescence microscope (IX51; Olympus). The targeting efficiency of the microbubbles to the ECV304 cells was assessed using a flow cytometer (Beckman Coulter). Following ultrasound irradiation, cell viability was determined by 0.4% trypan blue staining (Sigma) and the cell counting method.

### Determination of the in vivo targeting ability of ICAM-1-targeted microbubbles for the infarcted myocardium

All experiments in this section were carried out in accordance with the National Institutes of Health guide for the care and use of laboratory animals (NIH Publications no. 8023, revised in 1978). Approval from the Institutional Animal Care and Use Committee of the Wuhan University Health Science Centre (Wuhan, China) was also obtained to perform the experiments outlined below.

### Experimental groups

Healthy purebred New Zealand white rabbits (male or female, weighing 2.5–3.5 kg) were provided by the Wuhan Institute of Biology (Wuhan, China). In total, 15 rabbits were randomly divided into 3 groups as follows: i) the acute myocardial infarction (AMI) group, n=5: the rabbits with AMI received an injection of 1 ml anti-ICAM-1-targeted microbubbles through the ear vein and exposed to ultrasound irradiation; ii) the normal control group (n=5): healthy (normal) rabbits received an injection of 1 ml anti-ICAM-1-targeted microbubbles through the ear vein and exposed to ultrasound irradiation; and iii) the blank control group (n=5): rabbits with AMI did not receive a microbubble injection or were exposed to ultrasound irradiation for the exclusion of auto-fluorescence.

### Establishment of model of AMI

After being anesthetized with 20% urethane (dose of 5 ml/kg; Sigma) by ear vein injection and an electrocardiogram (ECG) monitor was connected, the heart was exposed from the left sternal border while the rabbit was spontaneously breathing, and the left circumflex branch of the coronary artery was ligated 5 mm from the left atrial appendage. Following ligation, the contraction of the regional myocardium was decreased and the infarcted myocardium turned pale. In this study, we regarded the ST segment elevation of the ECG (arched upward) of >2 mm as the sign of the successful establishment of the model of AMI.

### Microbubble transfer and ultrasound exposure

The microbubbles were injected through the ear vein of the rabbits. The iE33 ultrasound diagnostic platform (Philips Medical Systems HSG, Andover, MA, USA) and an M3S transducer (frequency 1–3 MHz) were used for ultrasound exposure. When the transthoracic echocardiogram was obtained in short axis at the mid-papillary muscle level, the ultrasound exposure commenced at the time point of microbubble injection, and was completed when the intra-myocardium microbubbles had completely vanished. The mechanical index was set as 1.3 and the overall gain was adjusted to 100% during the irradiation.

### Immunofluorescence

The myocardial tissue of the infarcted region was cut out and the sections were frozen immediately after microbubble transfer, and the green fluorescence intensity on the cardiac muscle vascular intima was observed under a fluorescence microscope to determine the adherence of the targeted microbubbles to the injured cardiac vascular intima. Hepatic and kidney tissue was also taken from the rabbits in order to determine whether any green fluorescence could be detected.

### Ang-1 gene transfection into the ischemic myocardium by UTMD using ICAM-1-targeted microbubbles

#### Experimental groups

In total, 30 rabbits with AMI were randomly divided into 4 groups and were subjected to Ang-1 gene therapy by UTMD: i) the ICAM-1-targeted microbubble group (TMB, n=8): the rabbits received an injection of the mixture of microbubble carrying ICAM-1 antibody and Ang-1 gene suspension under ultrasound exposure; ii) the non-TMB group (n=8): the rabbits only received an injection of microbubbles carrying the Ang-1 gene under ultrasound exposure; iii) the IMI group (n=8): the rabbits recevied a direct IMI of the Ang-1 gene plasmid under ultrasound exposure; and iv) the control group (n=6): the rabbits only received an injection of the Ang-1 gene plasmid intravenously under ultrasound exposure.

#### Gene transfection by UTMD

The Ang-1 gene plasmid was constructed by ligating the Ang-1 gene into the pcDNA3.1 vector with a cytomegalovirus promoter to drive Ang-1 expression. The SonoVue microbubble suspension was prepared as as described above. The suspension was mixed with 100 *µ*g of the Ang-1 plasmid at room temperature for 15 min, and was oscillated several times to ensure the sufficient contact of the plasmids and microbubbles. The rabbits were injected with 1 ml of the plasmid-microbubble solution (the gene concentration was 100 *µ*g/ml) through the ear vein, or were directly injected with 100 *µ*g of the plasmid at 5 points evenly distributed around the infarcted myocardium according to their grouping. Subsequently, ultrasound irradiation was applied for gene transfection.

The iE33 ultrasound diagnostic system and an M3S transducer were used for ultrasound exposure. The ‘contrast’ procedure was selected and the second harmonic mode was switched on. The probe emission frequency and the receiving frequency were 1.7 and 3.4 MHz, respectively, and the frame rate was 88 Hz. ECG-triggering was performed for every 4–8 cardiac cycles and the depth was set at 5 cm. Once the microbubble infusion commenced, the ultrasound beam was continuously delivered from the chest wall towards the heart of the rabbit. When the contrast agent was evenly distributed in the myocardium, the ultrasound blasting function was activated to explode the microbubbles. Ultrasound exposure persisted for 5 min with a mechanical index of 1.3 and 100% overall gain.

#### Echocardiography

A regular echocardiography examination was performed in all the groups on day 2 after AMI and 2 weeks following gene transfection. The regional wall motion was assessed, the left ventricular end-diastolic dimension (LVEDD), left ventricular ejection fraction (LVEF) and the infarct wall thickness of the left ventricular anterior wall (LVAW) were measured. The ΔEF was defined as [(LVEF post-treatment) - (LVEF pre-treatment)] and used to assess the changes in left ventricular cardiac function.

#### Myocardial perfusion

Myocardial contrast echocardiography (MCE) was performed on day 2 after AMI and 14 days following gene transfection to assess the infarcted myocardial perfusion which was described as uniform filling, partial filling and no filling (perfusion defect), as previously described (18). The MCE parameters were the same as those of the ‘contrast’ procedure mentioned above. The short axis view at the mid-papillary muscle level was obtained to observe the contrast agent filling and washout duration. All the acquired images were transferred to the workstation for off-line analysis frame by frame. The digital images were analyzed for the evaluation of the myocardial blood volume by calculating the contrast agent signal intensity ratio of the anterior wall (infarct area) to the posterior wall (normal area). The MCE images were analyzed using QLAB 6.0 software (Philips Medical Systems HSG).

#### Immunohistochemistry

Microvascular density (MVD; Factor VIII-positive structures) observed under a light microscope (BX51; Olympus) at x400 magnification was quantified immunohistochemically in the tissue sections of the infarcted myocardium which were obtained 2 weeks following gene transfection according the criteria of Weidner *et al* (19). Five visual fields with the highest number of capillaries were calculated to obtain the mean value. The left ventricle of the animal hearts was removed and stained with 3% Evans blue. The blue-stained area (non-ischemic) and the unstained area (ischemic) were separated and both were weighed. The ratio of the infarct area was expressed as the ratio of the weight of the infarcted myocardium/total myocardial weight for evaluating the infarct size.

#### Reverse transcription-polymerase chain reaction (RT-PCR)

Semi-quantitative RT-PCR (Thermo Scientific, Waltham, MA, USA) for the measuremnt of exogenous Ang-1 gene expression in the myocardium in all the groups was performed 2 weeks following transfection to determine the gene transfection efficiency. The Ang-1 primers were as follows: 5′-TGCCATTACCAGTCAGAGG-3′ (forward) and 5′-CAAGCATCAAACCACCATC-3′ (reverse); the PCR products were electrophoresed on a 1% agarose gel and stained with ethidium bromide. The mRNA expression levels of exogenous Ang-1 were normalized to the mRNA levels of β-actin.

#### Western blot analysis

Western blot analysis was used to the determine the Ang-1 protein expression level in the infarcted myocardium (which was normalized to the protein levels of β-actin) 2 weeks following gene transfection. Protein samples were separated by 10% sodium dodecyl sulfate-polyacrylamide gel electrophoresis (SDS-PAGE). The separated proteins were transferred onto polyvinylidene fluoride membranes and incubated with goat anti-Ang-1 antibody (ab133425; Abcam, Cambridge, UK) at 4°C overnight. The membranes were blocked with 5% non-fat milk and incubated with horseradish peroxidase-coupled mouse anti-goat IgG secondary antibodies (ab6789; Abcam) for 1 h at room temperature. The membranes were washed and exposed to X-ray film to detect the expression bands.

#### Statistical analysis

Variables were normally distributed and presented as the means ± standard deviation (SD). Differences between the data before and after gene transfection were analyzed using the Student’s t-tests. Comparisons among multiple stages were made using one-way ANOVA. All statistical tests were two-sided, and a p-value <0.05 was considered to indicate a statistically significant difference. All statistical analyses were performed using SPSS software version 19.0 (IBM SPSS, Chicago, IL, USA).

## Results

### Construction and appraisal of ICAM-1-targeted microbubbles

**Figure 1:**
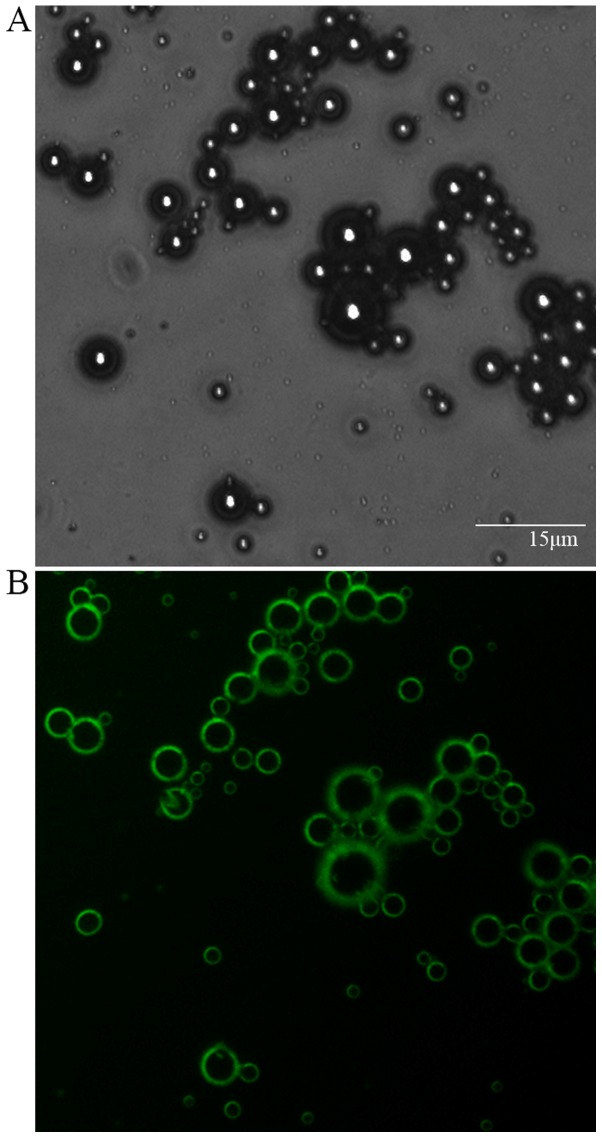
Intercellular adhesion molecule-1 (ICAM-1)-targeted microbubbles as observed under a light microscope and a fluorescence microscope to show the combination of FITC-labeled ICAM-1 antibody and SonoVue microbubbles. (A) Light microscope, x100 magnification. (B) Fluorescence microscope, x100 magnification.

As observed under an inverted microscope, the microbubbles were unevenly distributed and were partially aggregated in clusters (Fig. 1A). Following repeated and gently pipetting, the clustered microbubbles became dispersed again. Bright green fluorescence was detected at the fringe of the microbubbles under a fluorescence microscope (Fig. 1B), which demonstrated that the FITC-labeled ICAM-1 antibody bound to the surface of the microbubbles successfully. The flow cytometryshowed that the percentage of microbubbles which expressed green fluorescence in the mixed suspension was 52.3±5.3%.

### Targeting ability of the anti-ICAM-1-targeted microbubbles to inflammatory endothelial cells in vitro

**Figure 2:**
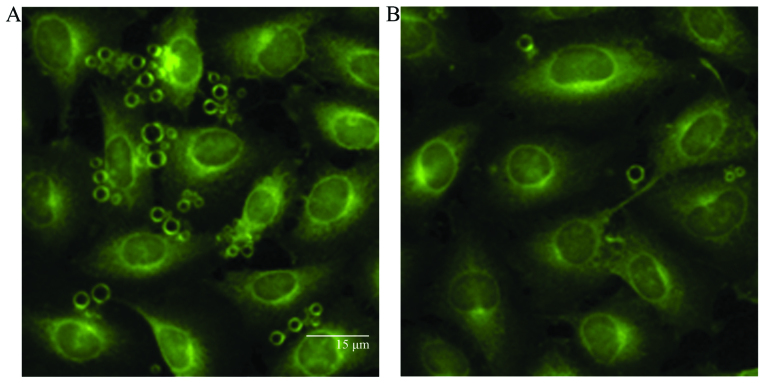
Combination of targeted microbubbles with ECV304 cells stimulated or not with interleukin-1β (IL-1β) as observed under a fluorescence microscope (x200 magnification). (A) The fringe of the IL-1β stimulated ECV304 cells showed bright green fluorescence. (B) Slight green fluorescence was observed at the fringe of the normal ECV304 cells not stimulated with IL-1β.

The fringe of the ECV304 cells expressed bright green fluorescence in the IL-1β-stimulated group (Fig. 2A). However, only slight green fluorescence was observed in the cells not stimulated with IL-1β; this fluorescence intenstiy was much less than that of the IL-1β-stimulated group (Fig. 2B). In the blank control, no green fluorescence was observed which excluded the influence of auto-fluorescence. In group 3, no green fluorescence was observed which indicated that the common microbubbles could not selectively adhere to the membrane of the IL-1β-stimulated cells despite the high expression of ICAM-1. The results indicated that the anti-ICAM-1-targeted microbubbles aggregated and adhered to the ECV304 cells which highly expressed ICAM-1. On the contrary, the anti-ICAM-1 targeted microbubbles did not adhere and aggregate to the ECV304 cells which did not express ICAM-1.

The combination efficiency of the ICAM-1-targeted microbubbles and the IL-1β-stimulated ECV304 cells was 86.7±5.6%, which was markedly higher than that of the microbubbles with the cells not stimulated with IL-1β (2.3±0.9%, p<0.01). The cell viability was 81.76±0.75, 83.04±1.05, 83.14±1.27, 82.54±1.32 and 93.06±0.94% in all 5 groups (groups 1–5, respectively). The viability of the cells in the blank control was higher than that of the cells in the other groups (p<0.01), while no significant differences were observed among the other 4 groups (p>0.05).

### Targeting ability of ICAM-1-targeted microbubbles for the infarcted myocardium in vivo

**Figure 3:**
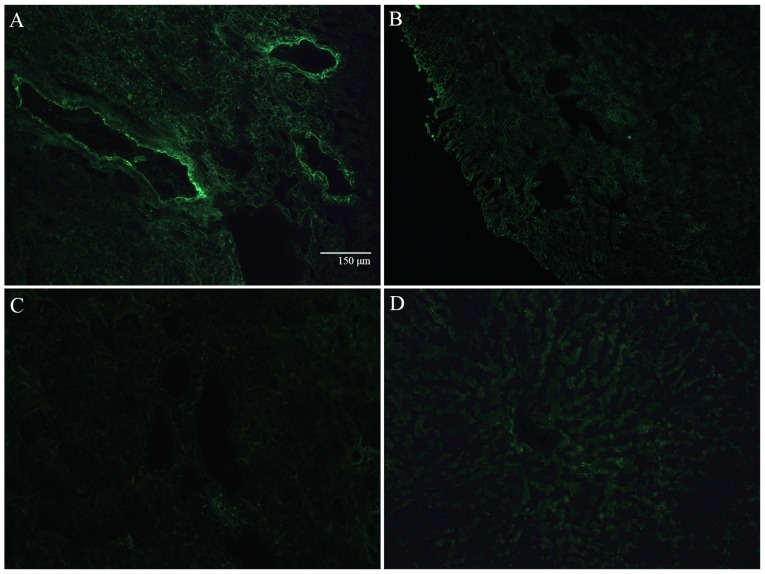
The adherence of targeted microbubbles carrying anti-intercellular adhesion molecule-1 (ICAM-1) antibody to injured vascular endothelium *in vivo* (fluorescence microscope, x200 magnification). (A and B) The fluorescence microscope images of the frozen section of cardiac muscle following injection of the targeted microbubbles into the rabbits with acute myocardial infarction (AMI) rabbit and in the normal rabbits, respectively. Only slight green fluorescence was observed in the normal myocardial vascular endothelium in the control group, which indicated that few ICAM-1-targeted microbubbles were released and had adhered at the normal vascular endothelium. (C) No green fluorescence was observed in the blank control group. (D) Frozen section of hepatic tissue from rabbits with AMI.

In the frozen sections, luminous green fluorescence was observed in the injured myocardial vascular endothelium of the infarcted myocardium in the experiment groups under a fluorescence microscope, which indicated that a large number of ICAM-targeted microbubbles was released and had adhered to the injured vascular endothelium (Fig. 3A). Only slight green fluorescence was observed in the normal myocardial vascular endothelium in the control group, which indicated that few ICAM-1-targeted microbubbles were released and had adhered at the normal vascular endothelium (Fig. 3B). Green fluorescence was not observed in the blank control group (Fig. 3C), which excluded the influence of auto-fluorescence of the infarcted myocardium. No green fluorescence was observed in the hepatic tissue (Fig. 3D).

### Ang-1 gene transfection into the ischemic myocardium by UTMD using ICAM-1-targeted microbubbles

After the model of AMI was successfully established, the regional wall motion was significantly reduced and the LVEF was decreased. At 2 weeks following gene transfection, compared to the baseline levels, LVEDD was decreased, while the LVEF and the infarct wall thickness were increased with varying degrees in all the animals apart from the controls. Furthermore, the LVEDD, LVEF and the infarct wall thickness in the TMB and IMI group were better than those of non-TMB group (Table I).

**Figure 4:**
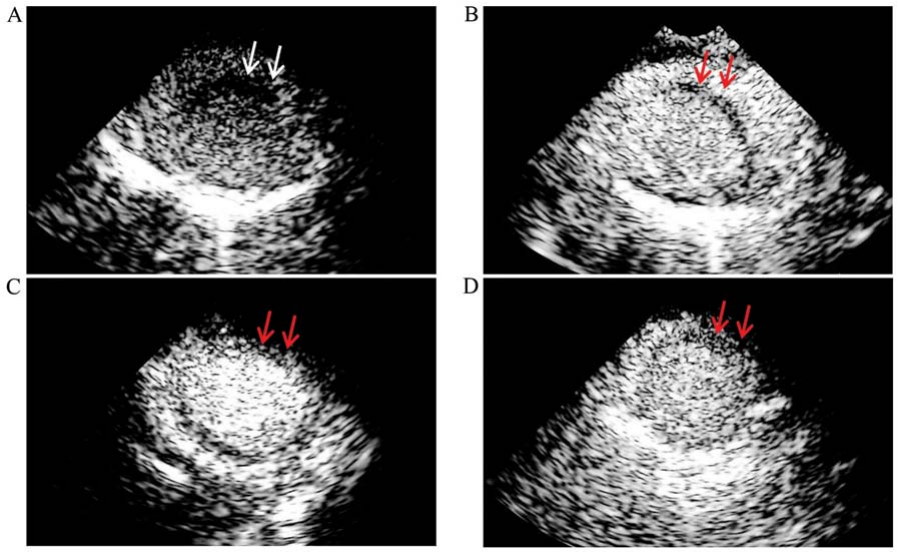
Myocardial perfusion of the infarct area in the control and gene-transfected animals. (A) Arrows indicate the contrast agent detection in the anterior wall 2 weeks after the induction of acute myocardial infarction (AMI) in the control rabbits. (B) Non-targeted microbubble delivery (TMB), (C) TMB and (D) intramyocardial injection (IMI) group rabbits; arrows indicate (B) slight filling or (C and D) partial filling of the contrast agent in the infarct area 2 weeks after gene transfection.

**Figure 5:**
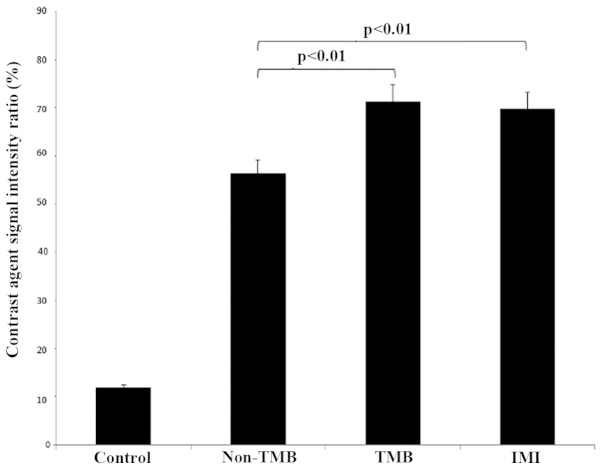
The contrast agent signal intensity ratio of the anterior wall to the posterior wall in the control and gene-transfected animals. p<0.01 indicates that the contrast agent signal intensity ratio of the anterior wall to the posterior wall in the targeted microbubble delivery (TMB) and intramyocardial injection (IMI) group was higher than that of the non-TMB group; no differences were observed between the TMB and IMI group.

MCE indicated that the contrast agent was obviously defective in the infarcted myocardium, while the filling of the other regions was satisfactory in the rabbits with AMI. At 2 weeks following transfection, the infarcted myocardium in the TMB and IMI group was partially filled as shown by MCE, while the infarct area of the animals in the non-TMB group was filled with less contrast agent (Fig. 4). The contrast agent signal intensity ratio of the anterior wall to the posterior wall in the TMB and IMI group was 0.71±0.04 and 0.70±0.08, respectively, which was significantly better than that of the non-TMB group (0.56±0.08, p<0.01) and the control group (0.12±0.02, p<0.01) (Fig. 5).

**Figure 6:**
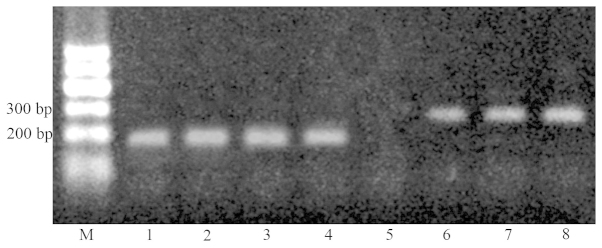
RT-PCR of angiopoietin-1 (Ang-1) mRNA expression in each group. No expression was observed in the control group, while gene expression was detected in the other 3 groups. Lanes 1-4, β-actin; lane 5, control; lane 6, non-targeted microbubble delivery (TMB) group; lane 7, TMB group; lane 8, intramyocardial injection (IMI) group.

**Figure 7:**
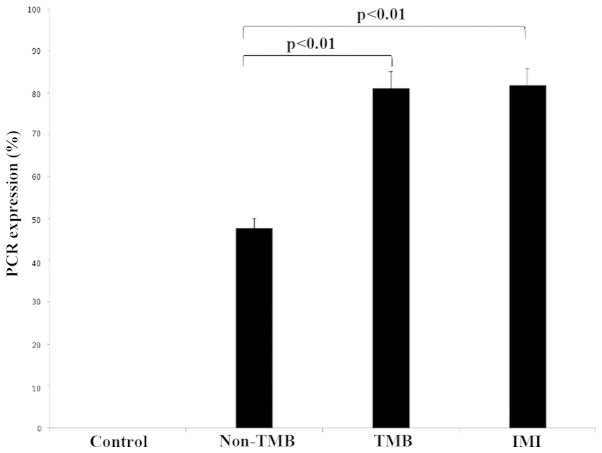
RT-PCR of angiopoietin-1 (Ang-1) mRNA expression in each group. No significant difference in gene expression was observed between the targeted microbubble delivery (TMB) and intramyocardial injection (IMI) group, while the expression in both these groups was significantly higher than that of the non-TMB group.

RT-PCR indicated that the relative expression of the Ang-1 gene in the myocardium was 0.48±0.03, 0.81±0.06 and 0.82±0.03 in the non-TMB, TMB and IMI group, respectively. The expression level in the non-TMB group was lower than that in the other 2 groups (p<0.01), and the expression level between the TMB and IMI group did not differ significantly (p=0.72). No obvious mRNA expression of Ang-1 was detected in the controls (Figs. 6 and 7).

**Figure 8:**
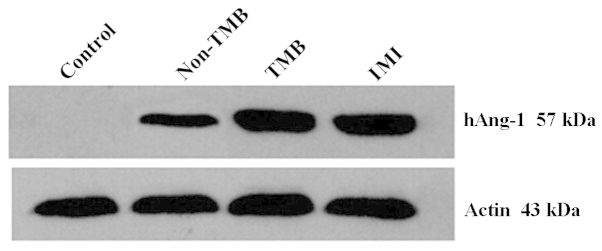
Western blot analysis of angiopoietin-1 (Ang-1) protein expression in each group. No significant levels of protein expression were detected in the control group. non-TMB, non-targeted microbubble delivery; TMB, targeted microbubble delivery; IMI, intramyocardial injection.

**Figure 9:**
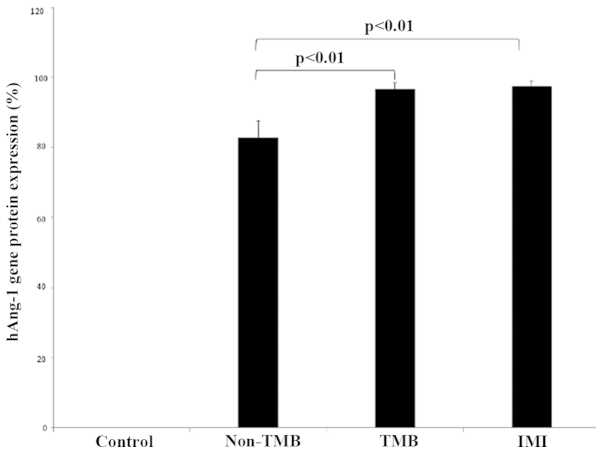
Western blot analysis of angiopoietin-1 (Ang-1) protein expression in each group. No significant difference in the protein level was observed between the targeted microbubble delivery (TMB) and intramyocardial injection (IMI) group, while the level in both these groups was significantly higher than that of the non-TMB group.

Ang-1 protein was detected in all the experimental animals apart from the controls by western blot analysis. The protein expression levels in the infarct area of the animals in the non-TMB, TMB and IMI group were 0.83±0.05, 0.96±0.02 and 0.97±0.02, respectively. The expression level in the TMB and IMI group was significantly higher than that in the non-TMB group (p<0.01); however, no statistically significant differences were observed in the expression level between the first 2 groups (p=0.83). N obvious protein expression was detected in the control group (Figs. 8 and 9).

**Figure 10:**
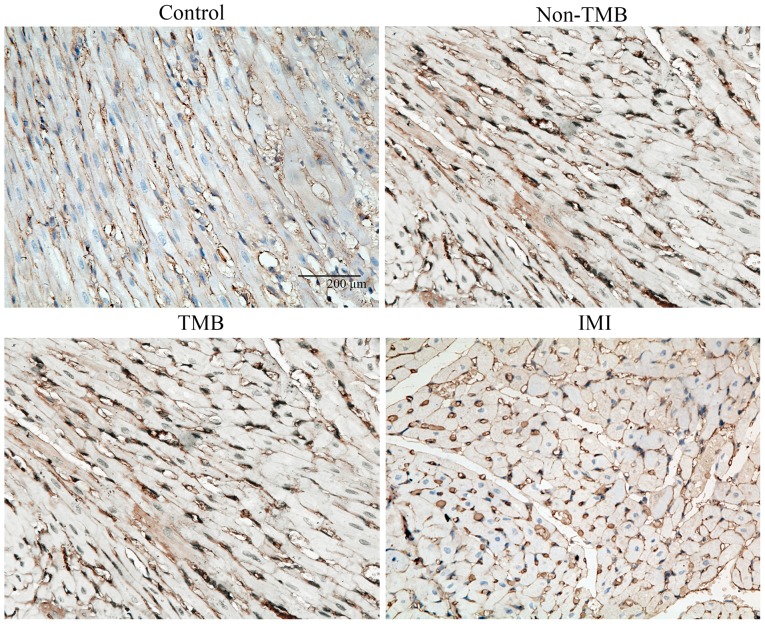
The microvessel density in the infarct area 2 weeks after gene transfection detected by immunohistochemistry. Only a small quantity of capillaries was observed in the controls; some capillaries were observed in the non-targeted microbubble delivery (TMB) group, and more capillaries in the TMB and intramyocardial injection (IMI) group.

**Figure 11:**
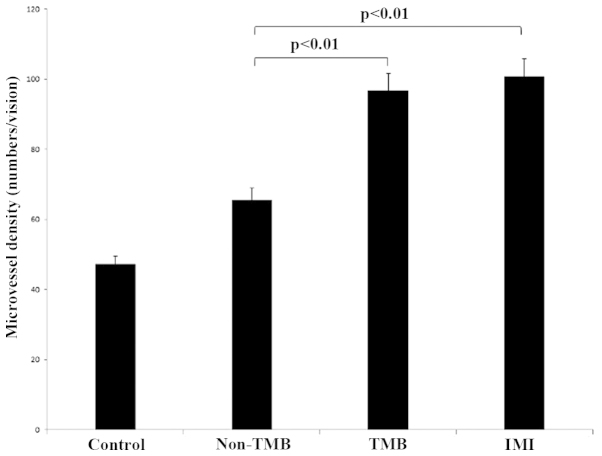
The number of capillaries in each group as detected by immunohistochemistry. The numbers of capillaries in the intramyocardial injection (IMI) group was higher than that in the other 3 groups (p<0.01, p<0.01 and p=0.028), the microvessel density in the targeted microbubble delivery (TMB) group was higher than that in the non-TMB group.

A greater number of factor VIII-positive structures was observed in the infarct area following gene transfection, apart from the control group. The MVD of the infarcted myocardium in the control group at 2 weeks after the induction of AMI was 51.07±4.83, which was lower than that of the other 3 groups (p<0.01). The MVD of the TMB group was (96.7±2.1) which was higher than that of the non-TMB group (65.6±4.4, p<0.01), but lower than that in the IMI group (100.7±3.6, p=0.028) (Figs. 10 and 11).

## Discussion

There is a high incidence and poor long-term outcome of ischemic heart disease both in developing and developed countries; thus, the treatment of refractory myocardial ischemia is of particularly importance. In recent years, gene-based therapeutic angiogenesis has been proven to be feasible in some pre-clinical and clinical studies (20). However, a good biosafety and high efficiency gene delivery system is still required for future clinical application (21). At present, UTMD, as a novel gene transfection technique, has shown its potential prosperity with clinical diagnostic ultrasound exposure within a recommended safety range. Ultrasound can produce and enhance bioeffects via acoustic cavitation, while the microbubbles can reduce the threshold of acoustic cavitation caused by ultrasound irradiation. Therefore, the combined application of ultrasound with microbubbles may be an effective and safe method for gene therapy. Although common microbubbles can deliver a gene to the target tissue by ultrasound exposure and achieve bioeffects, the transfer efficiency is not satisfactory as low numbers of microbubbles attach to the organ of interest, thus resulting in less gene uptake. A high gene transfection efficiency is considered to be related to a greater gene quantity and more microbubble destruction in the region of interest. It is known that naked DNA administered intravenously is easily degraded by DNase or captured by other cells before it reaches the target organ; thus increasing the DNA quantity in the region of interest is critical for improving the effects of gene therapy. Direct IMI can be used to achieve a high plasmid dose in the target tissue; however, this method of gene administration is not suitable for repeated application in clinical treatment. Therefore, in this study, we aimed to increase the quantity of the microbubbles in the infarcted myocardium using a target microbubble as a gene carrier, and as a consequence, greater microbubble destruction by ultrasound, leading to more gene intake. We progressively confirmed the feasibility and efficiency of the transfection of the exogenous angiogenesis gene to the infarcted myocardium by UTMD using microbubbles carrying anti-ICAM-1 antibody. First, we successfully constructed a ‘microbubble-anti-ICAM-1 monoclonal antibody’ complex by electrostatic attraction as a Ang-1 gene carrier and then found that this microbubble had better adhesion to inflammatory endothelial cells *in vitro* and the infarcted myocardium *in vivo* as compared with the non-targeted microbubbles. Moreover, we proved that the ICAM-1-targeted microbubbles are a more effective and feasible gene carrier for myocardial infarction gene therapy than common microbubbles, as their use increases the chance of the release of more bubbles to the area of interest and exerts better bioeffects, as determined by the standard of the effects of the direct IMI which is recognized as the most efficient method for exogenous gene transfection.

Previous studies have demonstrated a site orientation technique by connecting specific ligands to microbubbles, which gives the microbubbles the ability to recognize the lesion area of interest (22–25). Leong-Poi *et al* (26) prepared αV integrin-targeted microbubbles by connecting a monoclonal antibody to αV integrin on the surface. Ferrante *et al* (27) reported a dual-targeted phospholipid microbubble, which contained fluorocarbon gas internally and adhesion molecule P-selectin and vascular cell adhesion molecule-1 on the surface, and proved that microbubbles may be used for the detection of atherosclerotic plaque. It is known that ICAM is widely distributed on the surface of many cells, such as endothelial cells, and its expression level is relatively low under normal conditions. The expression of ICAM-1 is effected by various factors, such as IL-1, tumor necrosis factor (TNF), ischemia and hypoxia (28,29). The enhancement of ICAM-1 expression is a symbol of endothelial cell injury and leukocyte activation (30). Once ischemia occurs, multiple inflammatory factors induce the overexpression of ICAM-1 in microvascular endothelial cells. Furthermore, in injured endothelial cells, some inflammatory binding sites, such as the ICAM-1 spot are observed (31). Thus, in theory, targeted microbubbles carrying ICAM-1 antibodies would directionally transport more exogenous gene to the ischemic myocardium by selective combination with the injured endothelial cells expressing ICAM-1. To verify this point, we examined the adhesion ability of the ICAM-1-targeted microbubbles to IL-1β-stimulated ECV304 cells *in vitro* and the ischemic myocardium in rabbits with AMI *in vivo*. Our results revealed the feasibility of utilizing ICAM-1 antibody as a tissue-specific ligand for inflammatory cells, and compared with the non-targeted microbubbles, although in a complex internal environment, the targeted microbubbles remained stable, and still showed good adhesion to the ischemic myocardium.

A limitation of this study was the complex blood components and the sheer stress caused by blood flow *in vivo* which may weaken the intermolecular electrostatic adsorption capacity of the antibody and microbubbles, or the microbubbles themselves (32,33), which may reduce the targeting ability of ICAM-1-targeted microbubbles as compared to the level *in vitro*. Another limitation was the time window for the assessment of the bioeffects on angiogenesis. For better detecting the expression of the transfected gene, the time window was selected as 2 weeks following transfection, as previously described (34), although it was relatively short for ventricular remodeling. Therefore, the improvement of the regional function of the infarcted myocardium or heart function caused by therapeutic angiogenesis may have been more evident if the observation time was longer in our study.

In conclusion, our data demonstrate that SonoVue microbubbles carrying anti-ICAM-1 antibody provide a tissue-specific targeting strategy which leads to the release of a greatery quantity of microbubbles and higher gene levels into the infarcted myocardium. UTMD using ICAM-1-targeted microbubbles may significantly enhance the angiogenic effects of the Ang-1 gene than common microbubbles, which may become an outlet for the clinical application of gene therapy in the future.

## Figures and Tables

**Table I:** Evaluation of echocardiography before and after gene transfection (mean ± SD).

Group	Control	Non-TMB	TMB	IMI
n	6	8	8	8
LVEF (baseline)	0.50±0.05	0.51±0.04	0.52±0.05	0.50±0.02
LVEF (post)	0.51±0.04	0.67±0.05^a^	0.72±0.03^a^,^b^	0.72±0.05^a^,^b^
ΔEF	0.01±0.01	0.16±0.05^a^	0.20±0.05^a^,^b^	0.22±0.05^a^,^b^
LVEDD (baseline) (mm)	1.76±0.17	1.57±0.18	1.61±0.18	1.59±0.16
LVEDD (post) (mm)	1.73±0.19	1.39±0.16^a^	1.34±0.20^a^,^b^	1.28±0.19^a^,^b^
Δdimension (mm)	0.03±0.03	0.18±0.10^a^	0.26±0.06^a^,^b^	0.31±0.71^a^,^b^
LVAW (baseline) (mm)	1.82±0.15	1.91±0.16	1.96±0.20	2.09±0.24
LVAW (post) (mm)	1.82±0.08	2.09±0.12^a^	2.40±0.16^a^,^b^	2.43±0.20^a^,^b^
ΔLVAW (mm)	0.00±0.09	0.18±0.07^a^	0.44±0.16^a^,^b^	0.34±0.09^a^,^b^
p-value for LVEF	0.199	0.000	0.000	0.000
p-value for LVEDD	0.12	0.02	0.000	0.000
p-value for LVAW	1.000	0.000	0.000	0.000

Non-TMB, non-targeted microbubbles group; TMB, ICAM-1-targeted microbubbles group; IMI, intramyocardial injection group; LVEF, left ventricular ejection fraction; LVEDD, left ventricular end-diastolic dimension; LVAW, left ventricular anterior wall; LVEF, LVEDD, LVAW (baseline), LVEF, LVEDD, LVAW 2 days after AMI; LVEF, LVEDD, LVAW (post), LVEF, LVEDD, LVAW 2 weeks after gene transfection; ΔEF, (LVEF 2 weeks after gene transfection) - (LVEF 2 days after AMI); Δdimension, (LVEDD 2 weeks after gene transfection) - (LVEDD 2 days after AMI); ΔLVAW, (LVAW 2 weeks after gene transfection) - (LVAW 2 days after AMI).

a P<0.01 compared with the control group;

b P<0.01, compared with non-TMB group. p-value for LVEF, LVEDD, LVAW, the comparison of LVEF, LVEDD, LVAW between baseline and post.

## Abbreviations

UTMD: ultrasound-targeted microbubble destruction
ICAM-1: intercellular adhesion molecule-1
